# Evaluation of Potentially Avoidable Acute Care Utilization Among Patients Insured by Medicare Advantage vs Traditional Medicare

**DOI:** 10.1001/jamahealthforum.2022.5530

**Published:** 2023-02-24

**Authors:** Adam L. Beckman, Austin B. Frakt, Ciara Duggan, Jie Zheng, E. John Orav, Thomas C. Tsai, Jose F. Figueroa

**Affiliations:** 1 Harvard Medical School, Boston, Massachusetts; 2 Harvard Business School, Boston, Massachusetts; 3 US Department of Veterans Affairs, Boston Healthcare System, Boston University School of Public Health, Boston, Massachusetts; 4 Department of Health Policy and Management, Harvard T. H. Chan School of Public Health, Boston, Massachusetts; 5 Department of Surgery, Brigham and Women’s Hospital, Boston, Massachusetts; 6 Department of Medicine, Division of General Internal Medicine, Brigham and Women’s Hospital, Boston, Massachusetts

## Abstract

**Importance:**

Medicare Advantage plans have strong incentives to reduce potentially wasteful health care, including costly acute care visits for ambulatory care−sensitive conditions (ACSCs). However, it remains unknown whether Medicare Advantage plans lower acute care use compared with traditional Medicare, or if it shifts patients from hospitalization to observation stays and emergency department (ED) direct discharges.

**Objective:**

To determine whether Medicare Advantage is associated with differential utilization of hospitalizations, observations, and ED direct discharges for ACSCs compared with traditional Medicare.

**Design, Setting, and Participants:**

Cross-sectional study of US Medicare Advantage vs traditional Medicare beneficiaries from January 1 to December 31, 2018. Poisson regression models were used to compare risk-adjusted rates of Medicare Advantage vs traditional Medicare, controlling for patient demographic characteristics and clinical risk and including county fixed-effects. Data were analyzed between April 2021 and November 2022.

**Main Outcomes and Measures:**

Hospitalizations, observation stays, and ED direct discharges for ACSCs.

**Results:**

The study sample comprised 2 665 340 Medicare Advantage patients (mean [SD] age, 72.7 [9.8] years; 1 504 519 [56.4%] women; 1 859 067 [69.7%] White individuals) and 7 981 547 traditional Medicare patients (mean [SD] age, 71.2 [11.8] years; 4 232 201 [53.0%] women; 6 176 239 [77.4%] White individuals). Medicare Advantage patients had lower risk of hospitalization for ACSCs compared with traditional Medicare patients (relative risk [RR], 0.94; 95% CI, 0.93-0.95), primarily owing to fewer hospitalizations for acute conditions (eg, pneumonia). Medicare Advantage patients had a higher risk of ED direct discharges (RR, 1.44; 95% CI, 1.43-1.45) and observation stays (RR, 2.38; 95% CI, 2.34-2.41) for ACSCs vs traditional Medicare patients. Overall, Medicare Advantage patients were at higher risk of needing care for an ACSC (hospitalization, ED direct discharge, or observation stay) than traditional Medicare patients (RR, 1.30; 95% CI, 1.30-1.31). Within the Medicare Advantage population, patients in health maintenance organizations (HMOs) were at lower risk of ACSC-related hospitalization compared with patients in its preferred provider organizations (RR, 0.96; 95% CI, 0.95-0.98); however, those in the HMOs had a higher risk of ED direct discharge (RR, 1.08; 95% CI, 1.07-1.09) and observation stay (overall RR, 1.10; 95% CI, 1.02-1.12).

**Conclusions and Relevance:**

The findings of this cross-sectional study of Medicare Advantage and traditional Medicare patients with ACSCs indicate that apparent gains in lowering rates of potentially avoidable acute care have been associated with shifting inpatient care to settings such as ED direct discharges and observation stays.

## Key Points

**Question:**

How do rates of hospitalizations, emergency department (ED) direct discharges, and observation stays associated with ambulatory care−sensitive conditions compare between Medicare Advantage and traditional Medicare?

**Findings:**

This cross-sectional study of more than 10 million beneficiaries found that patients who experienced an ambulatory care−sensitive condition and were covered by Medicare Advantage were less likely to be hospitalized and more likely to be discharged directly from the ED or have an observation stay than were patients with traditional Medicare.

**Meaning:**

The findings of this cross-sectional study suggest that by shifting to other care settings, including ED direct discharges and observation stays, Medicare Advantage may be avoiding hospitalizations (acute care visits) for ambulatory care−sensitive conditions.

## Teaser

This cross-sectional study of 10 million Medicare beneficiaries compares hospitalizations, observation stays, and emergency department discharge rates between Medicare Advantage vs traditional Medicare to understand acute care utilization differences.

## Introduction

Given that approximately 25% of health spending in the US is estimated to be potentially wasteful,^1^ policy makers have prioritized identifying strategies to improve care and curb avoidable costs. Medicare Advantage was implemented with this ambition as a private managed care option using capitated monthly payments, rather than traditional Medicare fee-for-service payments. Medicare Advantage plans may better address wasteful spending compared with traditional Medicare by reducing acute care use for ambulatory care−sensitive conditions (ACSC)—conditions that may be avoidable when patients have adequate access to high-quality outpatient ambulatory care.^2^ However, it is unclear whether there are fewer potentially avoidable hospitalizations associated with Medicare Advantage compared with traditional Medicare. Prior studies have had mixed results^3,4,5,6,7,8,9,10,11,12^ or have been limited by narrow geographic scopes^7,10^ and have relied on data collected before the release of Medicare Advantage encounter data.^7,8,9,10,11,12^

Furthermore, emerging evidence has raised concerns that a reduction in hospitalizations may be an artifact of shifting sites of acute care (eg, observation stays, direct discharges from the emergency department [ED]), rather than an advantage of delivering better care in the ambulatory setting.^13,14^ The extent to which this shift is occurring within Medicare Advantage—and with what frequency vs traditional Medicare—is unknown. Given that Medicare Advantage is known to use prior authorizations and aggressive case management,^15^ these plans may perform better with potentially avoidable hospitalization measures not because of higher quality outpatient ambulatory care but instead owing to shifting patients toward observation status or ED direct discharges. In some cases, site shifting may be appropriate, whereas in others, it may be inappropriate; how it affects care quality is unclear. Moreover, certain types of Medicare Advantage plans (ie, health maintenance organizations [HMOs]) may be more likely to engage in this behavior than other types (ie, preferred provider organizations [PPOs]). Additionally, because Medicare Advantage covered 42% of the Medicare population in 2021 and enrollment is expected to continue growing,^16^ it is critical to understand whether possible differences in potentially avoidable hospitalizations between Medicare Advantage and traditional Medicare are explained by the shift in acute care sites.

Therefore, using national Medicare Advantage and traditional Medicare data, this study sought to determine whether Medicare Advantage may be associated with lower rates of potentially avoidable hospitalizations for ACSCs compared with traditional Medicare; whether Medicare Advantage may be associated with higher rates of observation status and/or ED direct discharges for the same ACSCs compared with traditional Medicare; and whether certain types of Medicare Advantage plans (eg, HMOs vs PPOs) are more likely to shift patients to observation status and/or ED direct discharge.

## Methods

The study was reviewed by the institutional review board of the Harvard T. H. Chan School of Public Health. Because the study was not considered to be human participants research, informed consent by study participants was waived. The study followed the Strengthening the Reporting of Observational Studies in Epidemiology (STROBE) reporting guidelines.

### Study Design and Data Collection

The study used a 20% sample of administrative claims and encounter data for patients enrolled in traditional Medicare (inpatient and outpatient files) and Medicare Advantage (encounter file) in 2018. The Medicare Beneficiary Summary File was used to obtain patient demographic information, including age, sex, race and ethnicity, dual-eligibility status for Medicaid, and residential zip code. Because the Medicare Payment Advisory Commission (MedPAC) has noted concerns regarding the completeness of pre-2018 Medicare Advantage encounter data,^17^ we used an approach validated by Jung and colleagues^18^ to limit the main analyses to Medicare Advantage contracts with highly reliable and complete data. Shown to be similar to the entire Medicare Advantage population,^19^ the list was developed by Jung and colleagues^18^ who identified highly reliable contracts by cross-checking Medicare Advantage encounter data with other external data sources, such as the MedPAR file (Medicare Provider Analysis and Review maintained by Centers for Medicare & Medicaid Services [CMS]) and HEDIS (Healthcare Effectiveness Data and Information Set). We used this list for our study (more details are available in the eMethods in Supplement 1).

We assigned a CMS Hierarchical Condition Category (HCC) risk score to each patient. The HCC is a marker of patient severity that is used by CMS to determine payments to Medicare Advantage plans. For those covered by traditional Medicare, the HCC score is based on all of the patient’s part A and B claims in the inpatient and outpatient files, and for those with Medicare Advantage, the score is based on all of the patient’s encounters in the inpatient and outpatient files. Medical record review claims from the Medicare Advantage encounter records were excluded from HCC calculations because of the risk of inflated coding. Prior literature^20,21,22^ has expressed concern regarding a more aggressive (ie, inflated) HCC risk code capture in Medicare Advantage plans compared with traditional Medicare. Given that CMS applies a 5.91% deflation of risk scores across Medicare Advantage when determining payments,^21^ the primary models in our analysis used a 6% reduction for each Medicare Advantage patient to account for differences in coding intensity. Because recent work^20^ suggests that Medicare Advantage plans generate 6% to 16% higher risk scores than traditional Medicare, we performed sensitivity analyses using 11% deflation (ie, middle point of the range in the literature^20,21,22^).

To identify ACSCs, we used the Prevention Quality Indicator algorithm developed by the Agency for Healthcare Research and Quality.^2^ The ACSCs admissions included acute and chronic conditions that are potentially preventable when an individual has access to high-quality ambulatory care. Chronic ACSCs included admission for 8 conditions: (1) asthma in an adult; (2) chronic obstructive pulmonary disease (COPD); (3) hypertension; (4) heart failure; (5) short-term diabetes complications (eg, ketoacidosis, hyperosmolarity disorders); (6) long-term diabetes complications, ie, involving the kidney, eye, or the neurologic or circulatory system; (7) uncontrolled diabetes; and (8) angina treated without procedure. Lower-extremity amputation among patients with diabetes was excluded because it was a rare outcome and is not amenable to ED direct discharge or observation stay. Acute ACSCs included admissions for 4 conditions: (1) dehydration, (2) bacterial pneumonia, (3) urinary tract infection, and (4) perforated appendix. The same algorithm was used to identify when the listed conditions shifted to observation stays (ie, coded as observation stays but not as admitted inpatient stays) or ED direct discharges (ie, patients who were treated and discharged directly from the ED).

### Primary and Secondary Outcomes

Primary outcomes were patient-level ACSC-associated hospitalizations, observation stays, and ED direct discharges in 2018 across 3 aggregated categories: the 8 chronic ACSCs combined, the 4 acute ACSCs combined, and the 12 ACSCs (both acute and chronic ACSCs) combined. As secondary outcomes, we examined each individual ACSC separately.

### Statistical Analysis

The demographic characteristics of patients with Medicare Advantage were compared with those of traditional Medicare patients using standardized mean differences (SMD).^23^ We also assessed differences in discharge locations (eg, home, transfer, skilled nursing facility) between Medicare Advantage vs traditional Medicare patients for observation stays and ED direct discharges. Given that the outcomes were number counts, we performed a Poisson regression model to determine the adjusted difference in the number of potentially avoidable hospitalizations for Medicare Advantage vs traditional Medicare patients.

The primary predictor was Medicare insurance type (Medicare Advantage vs traditional Medicare). Covariates included patient demographic characteristics (age, sex, dual-eligibility status for Medicaid, and self-reported race and ethnicity) and the number of months alive for each beneficiary. Patients were considered to be “dual-eligible for Medicaid” if they had qualified for Medicaid for at least 1 month during the study year.

Self-reported race and ethnicity data were categorized according to the Research Triangle Institute race code^24^ as Black, Hispanic, White, or other (American Indian/Alaska Native, Asian/Pacific Islander, and all other responses) and were included as covariates. Prior literature^13^ has documented higher rates of acute care visits associated with ACSCs among Black and Hispanic Medicare patients. These higher rates are likely owing to differences in access and barriers to high-quality ambulatory care, stemming from systemic and structural racism. Of note, the Agency for Healthcare Research and Quality intended ASCSs to be a quality measure aggregating at the level of a region; for this reason, this study’s analyses were limited to comparing patients at the county level, rather than at the clinician level. Therefore, we included county fixed-effects to account for within-county correlation and to eliminate between-county differences. We also included HCC risk scores as markers of patient severity.

Next, we repeated similar models for hospitalizations, observation stays, and ED direct discharges associated with acute ACSCs in aggregate, chronic ACSCs in aggregate, and each of the individual conditions separately. Then, we repeated a similar model that combined hospitalizations, observation stays, and ED direct discharges as the outcome across both acute and chronic conditions. Finally, we repeated the primary models to determine the adjusted differences in the number of episodes among Medicare Advantage patients in HMOs vs Medicare Advantage patients in PPOs.

Statistical tests were 2-tailed, and after Bonferroni adjustments for 12 primary outcomes and 32 secondary outcomes, *P* values < .004 were considered significant for primary outcomes and *P* values < .00156 for secondary outcomes. Data analyses were performed from April 2021 to November 2022 using SAS, version 9.4 (SAS Institute).

### Sensitivity Analyses

We performed a series of sensitivity analyses to assess the robustness of the study results. First, for better interpretability, we repeated the models using linear regression to determine the adjusted relative difference in the number of potentially avoidable hospitalizations between Medicare Advantage and traditional Medicare patients. Second, we repeated the primary Poisson regression models, adjusting for an 11% deflation of Medicare Advantage HCC risk scores, as noted previously.^20^ Third, we repeated the primary models to determine the adjusted differences in the number of episodes among Medicare Advantage patients in high-quality (ie, 4-5 star) plans vs patients in lower-quality (ie, 1-3 star) plans. Fourth, we limited the primary analysis to counties with more than 100 patients to assess for incidental parameters problems. Fifth, we accounted for overdispersion in the Poisson model. Sixth, we included the number of months alive as an offset instead of as a covariate. Seventh, we repeated the primary models to evaluate whether patterns were consistent across the 4 geographic regions of the US.

## Results

### Patient Characteristics

The study sample comprised 2 665 340 Medicare Advantage patients (mean [SD] age, 72.7 [9.8] years; 1 160 821 [43.6%] men and 1 504 519 [56.4%] women; 348 271 [13.1%] Black, 322 153 [12.1%] Hispanic, 1 859 067 [69.7%] White, and 135 849 [5.1%] other individuals) and 7 981 547 traditional Medicare patients (mean [SD] age, 71.2 [11.8] years; 3 749 346 [47.0%] men and 4 232 201 [53.0%] women; 737 365 [9.2%] Black, 549 047 [6.9%] Hispanic, 6 176 239 [77.4%] White, and 518 775 [6.5%] other individuals) (Table 1). All study patients were continuously enrolled during 2018 in a highly reliable Medicare Advantage contract or traditional Medicare. The 2 665 340 Medicare Advantage patients represented 67% of its beneficiaries.

**Table 1.** Patient Characteristics for Medicare Advantage vs Traditional Medicare

Patient characteristics	MA, %	TM, %	SMD
Patients, No.	2 665 340	7 981 547	NA
Age, mean (SD)	72.7 (9.8)	71.2 (11.8)	0.14
Female sex	56.4	53.0	0.07
Male sex	43.6	47.0	0.07
Race and ethnicity			
Black	13.1	9.2	0.13
Hispanic	12.1	6.9	0.19
White	69.8	77.4	0.18
Other^a^	5.1	6.5	0.06
Dual-eligible for Medicaid	14.1	12.7	0.04
Mean (SD) HCC risk score	0.86 (0.93)	0.88 (1.09)	0.02
US region			
Northeast	3.4	6.3	0.12
Midwest	16.6	18.5	0.05
South	20.6	22.2	0.04
West	43.9	38.9	0.10
Rural residence	18.9	20.4	0.04
County MA penetration, %	37.7	31.0	0.52
Area deprivation index, quartile			
Lowest	53.3	55.9	0.05
Second	24.1	21.2	0.07
Third	15.1	14.8	0.01
Highest	5.0	6.2	0.05

Abbreviations: HCC, hierarchal condition category; MA, Medicare Advantage; SMD, standardized mean difference (<0.1 is considered negligible); NA, not applicable; TM, traditional Medicare.

^a^ Including American Indian/Alaska Native, Asian/Pacific Islander, and other categories from the Research Triangle Institute race code.

The patients in highly reliable Medicare Advantage contracts had demographic characteristics similar to those of patients in the excluded Medicare Advantage plans (details are available in eTables 1 and 2 in Supplement 1). The Medicare Advantage and traditional Medicare patients also had similar demographic characteristics, ie, sex, dual-eligibility for Medicaid, mean HCC risk score, rural residence, and area deprivation index. However, on average, Medicare Advantage patients were older than traditional Medicare patients (mean [SD] age, 72.7 [9.8] years vs 71.2 [11.8] years; SMD, 0.14) and a greater proportion were Black individuals (13.1% vs 9.2%; SMD, 0.13). Discharge location after ED direct discharge or observation stay did not appear to differ substantially between Medicare Advantage and traditional Medicare patients (eTable 12 in Supplement 1).

### Hospitalizations for ACSCs

**Figure 1.**
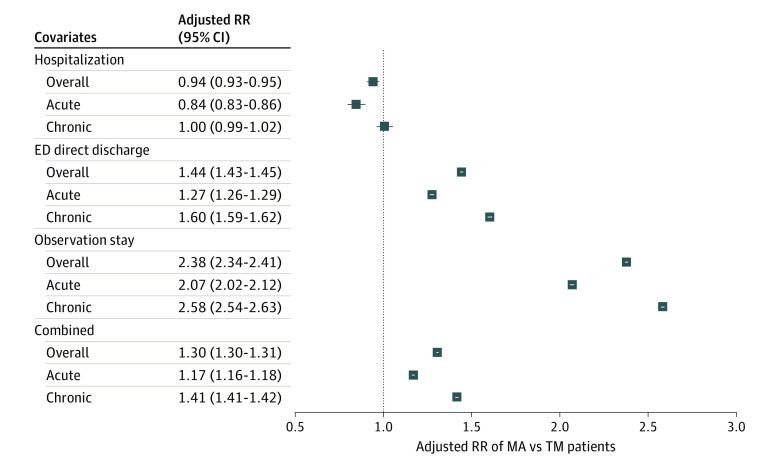
Differences in Avoidable Acute Care Episodes for Ambulatory Care−Sensitive Conditions Among Patients With Medicare Advantage vs Traditional Medicare Coverage Relative risks (RR) reflect estimates from Poisson models. The HCC risk scores were deflated by 6% in all models for all MA beneficiaries to account for more inflated coding practices. Each square illustrates the adjusted RR (95% CI) of MA patients, with TM patients serving as the reference. Analyses have been adjusted for age, sex, dual-eligibility status for Medicaid, self-reported race and ethnicity, the number of months alive for each beneficiary, and HCC risk score, including county fixed-effects. For the 12 primary outcomes, a Bonferroni adjustment was used assessing for *P* < .004. All coefficients met this threshold for statistical significance except hospitalization for chronic conditions. HCC refers to hierarchal condition category; MA, Medicare Advantage; and TM, traditional Medicare.

Medicare Advantage patients were less likely than traditional Medicare patients to be hospitalized for ACSCs, with a risk-adjusted relative risk (RR) of 0.94 (95% CI, 0.93-0.95; *P* < .001; Figure 1). This difference was associated with a relatively lower risk of hospitalization for acute ACSCs (RR, 0.84; 95% CI, 0.83-0.86; *P* < .001); however, no significant difference was found for chronic ACSCs (RR, 1.00; 95% CI, 0.99-1.02). The lowest RR among the individual acute ACSCs (Table 2) was observed for urinary tract infections (RR, 0.77; 95% CI, 0.75-0.79; *P* < .001). Among the chronic ACSCs, 3 diabetes-related complication measures had a significantly higher RR of hospitalization in Medicare Advantage compared with traditional Medicare, including diabetes short-term complications (RR, 1.13; 95% CI, 1.07-1.20; *P* < .001), and diabetes long-term complications (RR, 1.26; 95% CI, 1.22-1.30; *P* < .001).

**Table 2.** Differences in Avoidable Acute Care Episodes by Individual Ambulatory Care−Sensitive Conditions Between Medicare Advantage vs Traditional Medicare

Episode type	Unadjusted, mean (SD)^a^		Adjusted RR^b^ (MA vs TM)	*P* value
	Medicare Advantage	Traditional Medicare		
Patients, No.	2 665 340	7 981 547	NA	NA
**Hospitalization**				
Acute condition				
Perforated appendix	0.19 (14.09)	0.23 (15.50)	0.93 (0.84-1.03)	.18
Dehydration	3.16 (59.44)	4.63 (71.84)	0.84 (0.82-0.86)	<.001^c^
Community-acquired pneumonia	4.04 (67.09)	5.71 (79.60)	0.91 (0.89-0.93)	<.001^c^
Urinary tract infection	2.99 (61.44)	4.50 (72.59)	0.77 (0.75-0.79)	<.001^c^
Chronic condition				
Diabetes complications, short-term	0.71 (35.02)	0.84 (44.93)	1.13 (1.07-1.20)	<.001^c^
Diabetes complications, long-term	1.90 (54.38)	2.07 (55.80)	1.26 (1.22-1.30)	<.001^c^
COPD/asthma in older adults	5.46 (90.67)	6.56 (103.06)	1.02 (1.00-1.04)	.03
Hypertension	1.14 (35.82)	1.28 (38.77)	0.83 (0.80-0.87)	<.001^c^
Heart failure	9.78 (129.09)	13.10 (150.13)	1.00 (0.99-1.02)	.72
Uncontrolled diabetes	1.15 (38.45)	1.35 (42.48)	1.00 (0.96-1.05)	.93
Asthma in younger adults	0.02 (5.41)	0.04 (9.08)	1.27 (0.93-1.74)	.14
LEA in patients with diabetes, rate	0.58 (27.86)	0.70 (29.99)	1.29 (1.21-1.37)	<.001^c^
**ED direct discharge**				
Acute condition				
Perforated appendix	0.07 (8.75)	0.06 (7.62)	1.35 (1.12-1.62)	.002
Dehydration	4.65 (73.64)	3.90 (65.64)	1.38 (1.35-1.41)	<.001^c^
Community-acquired pneumonia	4.63 (75.70)	4.31 (69.85)	1.34 (1.31-1.37)	<.001^c^
Urinary tract infection	16.52 (154.44)	13.93 (138.32)	1.23 (1.21-1.24)	<.001^c^
Chronic condition				
Diabetes complications, short-term	0.18 (16.07)	0.14 (16.14)	2.22 (1.97-2.49)	<.001^c^
Diabetes complications, long-term	1.50 (49.40)	0.93 (44.65)	2.11 (2.02-2.21)	<.001^c^
COPD/asthma in older adults	11.62 (183.75)	8.53 (145.66)	1.64 (1.62-1.67)	<.001^c^
Hypertension	11.24 (126.40)	7.07 (97.91)	1.43 (1.41-1.45)	<.001^c^
Heart failure	5.51 (95.37)	3.90 (81.61)	1.88 (1.84-1.92)	<.001^c^
Uncontrolled diabetes	5.15 (99.17)	3.59 (82.29)	1.65 (1.61-1.68)	<.001^c^
Asthma in younger adults	0.14 (30.48)	0.22 (31.18)	1.65 (1.46-1.86)	<.001^c^
LEA in patients with diabetes, rate	0 (0)	0 (0)	1.00 (0-0)	>.99
**Observation stay**				
Acute condition				
Perforated appendix	0.04 (6.60)	0.03 (5.37)	1.54 (1.21-1.97)	<.001^c^
Dehydration	1.60 (43.02)	0.99 (32.33)	1.95 (1.87-2.03)	<.001^c^
Community-acquired pneumonia	0.95 (33.57)	0.50 (22.74)	2.27 (2.15-2.39)	<.001^c^
Urinary tract infection	2.03 (49.09)	1.07 (33.70)	2.09 (2.02-2.17)	<.001^c^
Chronic condition				
Diabetes complications, short-term	0.09 (10.96)	0.04 (6.64)	3.54 (2.94-4.26)	<.001^c^
Diabetes complications, long-term	0.53 (26.20)	0.17 (14.25)	4.21 (3.87-4.58)	<.001^c^
COPD/asthma in older adults	2.86 (66.46)	1.36 (41.99)	2.51 (2.43-2.59)	<.001^c^
Hypertension	2.22 (51.79)	0.84 (29.70)	2.47 (2.38-2.57)	<.001^c^
Heart failure	2.70 (62.15)	1.19 (41.57)	2.79 (2.69-2.88)	<.001^c^
Uncontrolled diabetes	1.00 (35.75)	0.50 (23.80)	2.27 (2.15-2.40)	<.001^c^
Asthma in younger adults	0.02 (6.74)	0.02 (4.94)	2.56 (1.72-3.81)	<.001^c^
LEA in patients with diabetes, rate	0 (0)	0 (0)	1.00 (0-0)	>.99

Abbreviations: COPD, chronic obstructive pulmonary disease; HCC, hierarchal condition category; LEA, lower-extremity amputation; MA, Medicare Advantage; NA, not applicable; RR, relative risks; TM, traditional Medicare.

^a^ The means reflect utilization per 1000 beneficiaries.

^b^ The RRs reflect estimates from Poisson models; HCC scores were deflated by 6% in all models for all MA beneficiaries to account for more inflated coding practices. Analyses were adjusted for age, sex, dual-eligibility status, self-reported race/ethnicity, number of months alive for each beneficiary, and HCC risk score, including county fixed-effects.

^c^ For the 32 secondary outcomes, a Bonferroni adjustment was used assessing for *P* <0.00156; these coefficients met this threshold for statistical significance.

### ED Direct Discharges and Observation Stays for ACSCs

Patients in Medicare Advantage were at higher risk of experiencing a direct discharge from the ED for both acute ACSCs (RR, 1.27; 95% CI, 1.26-1.29; *P* < .001) and chronic ACSCs (RR, 1.60; 95% CI, 1.59-1.62; *P* < .001) compared with patients in traditional Medicare. When examining individual ACSCs, the risk of ED direct discharge was higher among Medicare Advantage patients for every individual ACSC for traditional Medicare patients. Similarly, the risk of observation stay was consistently greater for Medicare Advantage patients than for traditional Medicare patients (overall RR, 2.38; 95% CI, 2.34-2.41; *P* < .001). Combining all types of episodes, patients in Medicare Advantage were at higher risk of needing care for any ACSC (hospitalization, ED direct discharge, or observation stay) than patients in traditional Medicare (overall RR, 1.30; 95% CI, 1.30-1.31*; P* < .001; acute RR, 1.17; 95% CI, 1.16-1.18*; P* < .001; chronic RR, 1.41; 95% CI, 1.41-1.42*; P* < .001).

### ACSCs in Medicare Advantage HMOs vs PPOs

**Figure 2.**
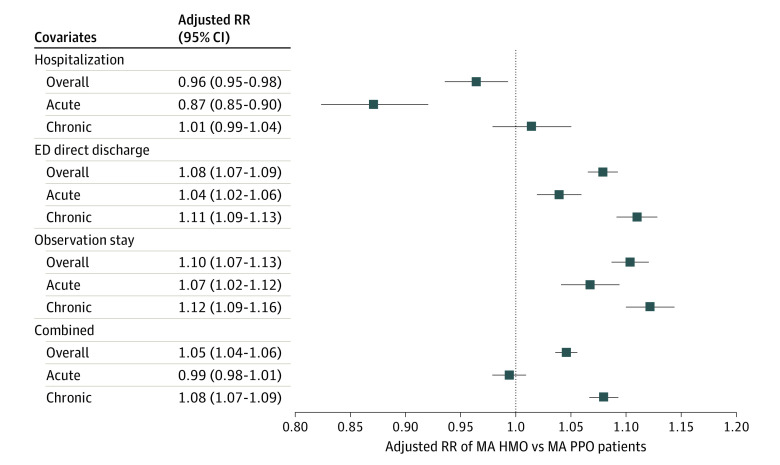
Differences in Avoidable Acute Care Episodes for Ambulatory Care−Sensitive Conditions Among MA HMOs vs MA PPOs Relative risks (RR) reflect estimates from Poisson models. Each square illustrates the adjusted RR (95% CI) of MA HMO patients, with MA PPO patients serving as the reference. Analyses have been adjusted for age, sex, dual-eligibility status for Medicaid, self-reported race and ethnicity, the number of months alive for each beneficiary, and HCC risk score, including county fixed-effects. For the 12 primary outcomes, a Bonferroni adjustment was used assessing for *P* < .004. All coefficients met this threshold for statistical significance except hospitalization for chronic conditions and combined for acute conditions. HCC refers to hierarchal condition category; HMO, health maintenance organization; MA, Medicare Advantage; and PPO, preferred provider organization.

Among the Medicare Advantage population, patients in Medicare Advantage HMOs were at lower risk of having a hospitalization for ACSCs compared with patients in Medicare Advantage PPOs (RR, 0.96; 95% CI, 0.95-0.98; *P* < .001); this may be explained by differences in acute ACSCs (RR, 0.87; 95% CI, 0.85-0.90; *P* < .001; Figure 2). For both acute and chronic ACSCs, Medicare Advantage HMOs had a higher risk of ED direct discharge (overall RR, 1.08; 95% CI, 1.07-1.09; *P* < .001) and observation stay (overall RR, 1.10; 95% CI, 1.02-1.12; *P* < .001). Across all types of episodes (hospitalization, ED direct discharge, and observation stay), patients in Medicare Advantage were at higher risk of needing care for an ACSC than patients in traditional Medicare (overall RR, 1.05; 95% CI, 1.04-1.06; *P* < .001) owing to differences in chronic ACSCs (RR, 1.08; 95% CI, 1.07-1.09; *P* < .001).

### Sensitivity Analyses

Results of sensitivity analyses were similar between Medicare Advantage and traditional Medicare when using linear regression models (eTable 3 and eTable 5 in Supplement 1), using a more aggressive 11% HCC risk score deflation for Medicare Advantage patients (eTable 4 in Supplement 1). For the 11% deflation analysis, the main difference was that risk of hospitalization for Medicare Advantage compared with traditional Medicare was lower for acute ACSCs (RR, 0.89; 95% CI, 0.88-0.90; *P* < .001), higher for chronic ACSCs (RR, 1.08; 95% CI, 1.06-1.09; *P* < .001), and no different overall (RR, 1.00; 95% CI, 0.99-1.01; *P* < .001). Across all episode types (hospitalization, ED direct discharge, and observation stay), patients in high-quality Medicare Advantage plans compared with lower-quality Medicare Advantage plans had slightly lower rates of needing care for an ACSC (overall RR, 0.95; 95% CI, 0.93-0.96; *P* < .001) (eTable 6 in Supplement 1). The primary results were similar, even after limiting counties with more than 100 patients (eTable 7 in Supplement 1), accounting for overdispersion (eTables 8 and 10 in Supplement 1), and including the number of months alive as an offset (eTable 9 in Supplement 1). The results were also similar across the 4 US geographic regions (eTable 11 in Supplement 1).

## Discussion

This national study of Medicare patients found differences in rates of potentially avoidable acute care visits between Medicare Advantage and traditional Medicare. Although Medicare Advantage patients were less likely to be hospitalized for ACSCs, they were more likely to have observation stays and ED direct discharges for the same conditions. These findings raise the possibility of a shifting effect, whereby the Medicare Advantage patients are more likely than traditional Medicare patients to be treated under observation status or in the ED instead of being admitted to the hospital. Combining number of hospitalizations, observation stays, and ED direct discharges, Medicare Advantage patients were more likely to have any acute episode of care for ACSCs than were traditional Medicare patients. Taken together, these observations suggest that apparent differences in potentially avoidable hospitalizations among Medicare Advantage plans may be explained by shifting inpatient care to other settings, such as ED direct discharges and observation stays.

The findings of this study have several potential policy, management, and practice explanations. First, the strong financial incentives that Medicare Advantage faces—inherent in capitated payments—to prioritize cost efficiency in care delivery may underlie these differences considering that admissions are generally more costly than ED direct discharges or observation stays. Therefore, Medicare Advantage plans may be more aggressive and effective than traditional Medicare in using tools, such as prior authorizations, to ensure that patients are treated by the lowest cost site of care allowed. This possibility is likely given that in 2018, 70% of Medicare Advantage enrollees were in a plan that required prior authorization for inpatient hospital stays, whereas traditional Medicare did not widely require this authorization.^8,15^ Site shifting may explain why Medicare Advantage had fewer admissions for acute ACSCs (eg, pneumonia or UTI) but not for chronic ACSCs (eg, heart failure exacerbations)—clinicians may characterize the former as more feasible to treat across settings than the latter. Second, Medicare Advantage plans may further influence utilization by affecting patient choices through more narrow clinician networks and decision-making (ie, payment incentives to promote efficiency), whereas traditional Medicare is less likely to use these levers.^5^ The possibility that site shifting in Medicare Advantage is occurring to promote more cost efficiency is further supported by our study’s finding that HMO plans were more likely than PPO plans to demonstrate shifting trends; generally, HMOs are more restrictive (eg, in network size or referral requirements) than PPOs.^25^

A third possibility is that Medicare Advantage patients are healthier at time of presentation than traditional Medicare patients, and therefore, receive better outpatient ambulatory care at an appropriately lower level of acute care, which avoids inpatient admission. However, even after accounting for differences in inflated coding practices, our study found that Medicare Advantage had higher aggregate rates of any hospitalization, observation stay, or ED direct discharge for an ACSC, which argues against the theory that Medicare Advantage patients are generally much healthier. Additionally, we found that hospitalizations for diabetes complications were higher among Medicare Advantage patients, a difference that may be associated with its financial disincentives to provide higher-cost diabetes therapies in the outpatient ambulatory care setting. For example, a recent study found^26^ that Medicare Advantage patients had a lower likelihood of receiving newly approved, evidence-based antihyperglycemic medications compared with traditional Medicare patients, likely owing to drug cost.

These study findings raise important questions regarding how these trends may affect patient finances and clinical outcomes. In traditional Medicare, patients under observation status often incur greater out-of-pocket (OOP) costs than patients who are hospitalized.^27^ It is possible that Medicare Advantage patients are also incurring higher OOP costs than those who are ultimately hospitalized, which could detrimentally affect patient finances. However, unlike traditional Medicare, Medicare Advantage plans do include a maximum on OOP spending, which may affect the decision to use more ED care. However, given that there is little data on the OOP costs of observation stays with Medicare Advantage,^28^ further research is needed to clarify how these utilization patterns affect patient finances.

Consequences for clinical quality are also currently unclear. For ED direct discharges, prior research has raised concerns that traditional Medicare may harm patients by inappropriately discharging them to home instead of allowing hospitalization.^29,30^ For observation stays, studies have suggested that traditional Medicare may harm patients by disallowing a beneficial postacute stay at a skilled nursing facility (ie, per traditional Medicare’s 3-night consecutive inpatient stay requirement).^27,28^ However, 2 additional factors are at play in Medicare Advantage—some of its plans have waived the 3-night requirement and its patients are more likely to go to lower-quality skilled nursing facilities^28,31^—meaning that the net effects of these trends in Medicare Advantage are not obvious. If shifting care to lower-cost settings is not negatively affecting quality or outcomes, this trend could reflect an important mechanism for cost savings. Overall, further research is needed to elucidate how these patterns affect long-term patient clinical trajectories, and ultimately clarify whether the potential cost efficiencies of site shifting are occurring alongside tradeoffs in care quality.

The findings of this study add to a growing body of literature that evaluates utilization and quality-of-care between Medicare Advantage and traditional Medicare patients. Other prior work^6,9,10,11^ has also found fewer hospitalizations for ACSCs in the Medicare Advantage program. Our study findings broaden the literature by observing greater use of ED direct discharges and observation stays for the same ACSCs among Medicare Advantage vs traditional Medicare patients at a national level, which may be the principal reason for lower use of hospitalizations with Medicare Advantage. Additionally, these findings suggest the need for caution in relying on hospitalizations for ACSCs to serve as an indicator of higher-quality care in ambulatory settings; it is currently used as a quality measure to assess performance in value-based care models, eg, Accountable Care Organizations and Medicare Advantage plans. At the very least, these study results suggest the need for this measure to be accompanied by careful monitoring of observation stays and ED direct discharges for the same conditions.

### Limitations

This study had several limitations. First, this study uses observational data, and thus cannot definitively conclude that the differences noted by Medicare insurance type are causally related. Second, there are concerns that Medicare Advantage encounter data may be incomplete. For this reason, this analysis was limited to a set of Medicare Advantage contracts with high data completeness that have been previously validated in other studies.^18,19^ Additionally, although one may expect Medicare Advantage to have fewer events in acute care settings given these concerns, this study reveals the opposite. Therefore, this study’s net differences may be underestimated when comparing with traditional Medicare. Third, there are concerns that Medicare Advantage plans use more inflated practices to capture risk severity. Although this study deflated individual risk scores in Medicare Advantage to account for these practices, it is possible that important differences in risk capture still exist. Again, however, one would expect Medicare Advantage beneficiaries to have had fewer total ACSC-related events if they were indeed healthier, which is not what this study found. Fourth, it is theoretically possible that differences between Medicare Advantage and traditional Medicare in accessibility of postacute care discharge locations (eg, access to a skilled nursing facility) contributed to the level of inpatient care that a patient received; however, our analyses did not reveal differences in discharge location between these populations. Fifth, this study did not assess other potential sites where ACSCs may present, including urgent care visits, in-person visits to primary care physicians, or remote outpatient visits; therefore, comparisons between rates of ACSCs in Medicare Advantage and traditional Medicare in those settings cannot be drawn. Finally, ACSCs are limited in their ability to study the quality of care of Medicare Advantage plans, with limitations including associations with nonclinical factors, eg, social vulnerability and economic deprivation.^32,33^ For this reason, we included county fixed-effects to limit analyses of beneficiaries by Medicare insurance within geographic areas of similar social vulnerability. Furthermore, this study cannot reliably assess which hospitalizations were necessary vs potentially wasteful, nor which encounters were appropriately triaged to observation status or directly treated and discharged from the ED. Other work has found that Medicare Advantage is no different nor does it outperform traditional Medicare^3,34,35^ when focused on other elements of quality (eg, measures of ambulatory care) and narrow subpopulations. Yet the active use of ACSCs by policy makers to assess Medicare Advantage quality (eg, star ratings) creates incentives for plans to reduce ACSCs, thereby making it important to study these measures. Although this study’s results should be interpreted in the context of the broader literature, its findings advocate for the careful monitoring of care quality among beneficiaries of Medicare Advantage.

## Conclusions

This cross-sectional study using national Medicare data found that apparent decreases in the number of potentially avoidable hospitalizations among Medicare Advantage vs traditional Medicare patients are occurring concurrent to the higher use of ED direct discharges, observation stays, and aggregate acute care episodes for the same conditions. Future research should aim to untangle how these trends are associated with the quality and efficiency of the care that patients receive with Medicare Advantage compared with traditional Medicare coverage.

## Notes

Supplement 1. **eMethods. **Supplemental methods **eTable 1. **Patient characteristics for Medicare Advantage contracts with highly reliable data versus Medicare Advantage excluded contracts **eTable 2. **Event characteristics for Medicare Advantage contracts with highly reliable data versus Medicare Advantage excluded contracts **eTable 3. **Differences in avoidable acute care episodes related to ambulatory care−sensitive conditions among Medicare Advantage versus Traditional Medicare using linear regression models **eTable 4. **Differences in avoidable acute care episodes related to ambulatory care−sensitive conditions among Medicare Advantage versus Traditional Medicare using 11% HCC deflation **eTable 5. **Differences in avoidable acute care episodes related to ambulatory care−sensitive conditions among MA HMOs versus MA PPOs using linear regression models **eTable 6. **Differences in avoidable acute care episodes related to ambulatory care−sensitive conditions among MA high-quality versus MA lower-quality plans **eTable 7. **Differences in avoidable acute care episodes related to ambulatory care−sensitive conditions among Medicare Advantage versus Traditional Medicare using Poisson models, limiting to counties with 100+ patients **eTable 8. **Differences in avoidable acute care episodes related to ambulatory care−sensitive conditions among Medicare Advantage versus Traditional Medicare using Poisson models, accounting for overdispersion **eTable 9. **Differences in avoidable acute care episodes related to ambulatory care−sensitive conditions among Medicare Advantage versus Traditional Medicare using Poisson models, accounting for overdispersion and with months alive as offset **eTable 10. **Differences in avoidable acute care episodes related to ambulatory care−sensitive conditions among Medicare Advantage versus Traditional Medicare using Poisson models, accounting for overdispersion and without HCC adjustment **eTable 11. **Differences in avoidable acute care episodes related to ambulatory care−sensitive conditions among Medicare Advantage versus Traditional Medicare using Poisson models, stratified by geographic region **eTable 12. **Discharge location after ED direct discharge or observation stay for Medicare Advantage versus Traditional Medicare

Supplement 2.
Data Sharing Statement
