# Error in Statistical Analysis

**DOI:** 10.1001/jamahealthforum.2023.1361

**Published:** 2023-06-02

In the original investigation titled “Evaluation of Potentially Avoidable Acute Care Utilization Among Patients Insured by Medicare Advantage vs Traditional Medicare,”^1^ published online on February 24, 2023, a statistical coding error slightly changed the denominator of the sample of Medicare Advantage and traditional Medicare beneficiaries. Corrections to percentages, point estimates, and confidence intervals did not affect the study findings or conclusions. This article was corrected online.
