# Supplementary material for: Evaluation of Potentially Avoidable Acute Care Utilization Among Patients Insured by Medicare Advantage vs Traditional Medicare
Source: JAMA Health Forum. 2023 Feb 24;4(2):e225530. doi: 10.1001/jamahealthforum.2022.5530 (PMC9958527; doi:10.1001/jamahealthforum.2022.5530)
Supplement: Supplement 1. — eMethods. Supplemental methods eTable 1. Patient characteristics for Medicare Advantage contracts with highly reliable data versus Medicare Advantage excluded contracts eTable 2. Event characteristics for Medicare Advantage contracts with highly reliable data versus Medicare Advantage excluded contracts eTable 3. Differences in avoidable acute care episodes related to ambulatory care−sensitive conditions among Medicare Advantage versus Traditional Medicare using linear regression models eTable 4. Differences in avoidable acute care episodes related to ambulatory care−sensitive conditions among Medicare Advantage versus Traditional Medicare using 11% HCC deflation eTable 5. Differences in avoidable acute care episodes related to ambulatory care−sensitive conditions among MA HMOs versus MA PPOs using linear regression models eTable 6. Differences in avoidable acute care episodes related to ambulatory care−sensitive conditions among MA high-quality versus MA lower-quality plans eTable 7. Differences in avoidable acute care episodes related to ambulatory care−sensitive conditions among Medicare Advantage versus Traditional Medicare using Poisson models, limiting to counties with 100+ patients eTable 8. Differences in avoidable acute care episodes related to ambulatory care−sensitive conditions among Medicare Advantage versus Traditional Medicare using Poisson models, accounting for overdispersion eTable 9. Differences in avoidable acute care episodes related to ambulatory care−sensitive conditions among Medicare Advantage versus Traditional Medicare using Poisson models, accounting for overdispersion and with months alive as offset eTable 10. Differences in avoidable acute care episodes related to ambulatory care−sensitive conditions among Medicare Advantage versus Traditional Medicare using Poisson models, accounting for overdispersion and without HCC adjustment eTable 11. Differences in avoidable acute care episodes related to ambulatory care−sensitive conditions amon [file jamahealthforum-e225530-s001.pdf]

Supplemental Online Content

Beckman AL, Frakt AB, Duggan C, et al. Evaluation of potentially avoidable acute care utilization among patients insured by Medicare Advantage vs Traditional Medicare. *JAMA Health Forum.* 2023;4(2):e225530. doi:10.1001/jamahealthforum.2022.5530

eMethods 1. Supplemental methods

eTable 1. Patient characteristics for Medicare Advantage contracts with highly reliable data versus Medicare Advantage excluded contracts

Table 2. Event characteristics for Medicare Advantage contracts with highly reliable data versus Medicare Advantage excluded contracts

eTable 3. Differences in avoidable acute care episodes related to ambulatory care-sensitive conditions among Medicare Advantage versus Traditional Medicare using linear regression models

eTable 4. Differences in avoidable acute care episodes related to ambulatory care-sensitive conditions among Medicare Advantage versus Traditional Medicare using 11% HCC deflation

eTable 5. Differences in avoidable acute care episodes related to ambulatory care-sensitive conditions among MA HMOs versus MA PPOs using linear regression models

eTable 6. Differences in avoidable acute care episodes related to ambulatory care-sensitive conditions among MA high-quality versus MA lower-quality plans

eTable 7. Differences in avoidable acute care episodes related to ambulatory care-sensitive conditions among Medicare Advantage versus Traditional Medicare using Poisson models, limiting to counties with 100+ patients

eTable 8. Differences in avoidable acute care episodes related to ambulatory care-sensitive conditions among Medicare Advantage versus Traditional Medicare using Poisson models, accounting for overdispersion

eTable 9. Differences in avoidable acute care episodes related to ambulatory care-sensitive conditions among Medicare Advantage versus Traditional Medicare using Poisson models, accounting for overdispersion and with months alive as offset

eTable 10. Differences in avoidable acute care episodes related to ambulatory care-sensitive conditions among Medicare Advantage versus Traditional Medicare using Poisson models, accounting for overdispersion and without HCC adjustment

eTable 11. Differences in avoidable acute care episodes related to ambulatory care-sensitive conditions among Medicare Advantage versus Traditional Medicare using Poisson models, stratified by geographic region

eTable 12. Discharge location after ED direct discharge or observation stay for Medicare Advantage versus Traditional Medicare

eMethods 1. Supplemental methodology

Identification of high-reliable MA contracts

The Medicare Payment Advisory Commission (MedPAC) has noted concerns about the completeness of MA encounter data from years prior to 2018. Therefore, we have followed a recently validated approach that identifies MA contracts with high reliability of completeness as performed by Jung et al.[18] This approach identifies highly reliable contacts by cross-checking MA encounter data with other external data sources, including the CMS Medicare Provider Analysis and Review (MedPAR) file and the Healthcare Effectiveness Data and Information Set (HEDIS) on the number of inpatient stays, ambulatory care visits, and ED visits. The list of highly reliable contracts were made available by Jung et al. In total, there were 210 contracts in 2018 MA Encounter data that were included in our analyses while 508 MA contracts were excluded. On average, there were 13,426 MA beneficiaries in each contract with highly reliable data vs. 3,427 MA beneficiaries in less reliable contracts. In eTable 1, we compare the characteristics of MA beneficiaries in highly reliable MA contracts vs. those in low reliable contracts. In eTable 2, we also compare the number of acute care visits across each type of episode in the highly reliable MA contracts vs. those in less reliable MA contracts.

Calculating risk scores

When calculating HCC scores, given potential concerns for upcoding, we limited our calculation of HCC risk scores to inpatient and outpatient files only for both MA and TM beneficiaries. We excluded records made from home health nursing visits and the carrier file as well, given potential concerns that these codes contribute to aggressive upcoding in MA.

Socioeconomic status

We used the Area Deprivation Index (ADI), a widely used measure of socioeconomic neighborhood disadvantage, based on the beneficiaries' zip code of residence. The ADI variable was downloaded from the Neighborhood Atlas website created by the University of Wisconsin School of Medicine and Public Health at the following link: https://www.neighborhoodatlas.medicine.wisc.edu/.

Star Ratings Analysis

For the sensitivity analysis examining adjusted differences in the number of episodes among MA patients in high-quality (i.e., 4-5 star) plans vs. patients in lower-quality (i.e, 1-3 star) plans, we identified star ratings using 2020 star ratings, which correspond to 2018 MA encounter data.[37] These models used Hospital Referral Region (HRR)-fixed effects instead of county-fixed effects to address sparsity of certain types of plans in some counties.

© 2023 Beckman AL et al. *JAMA Health Forum.*

eTable 1. Patient characteristics for Medicare Advantage contracts with highly reliable data versus Medicare Advantage excluded contracts

Patient Characteristics	Patients in Medicare Advantage highly reliable contracts (N=2,665,340)	Patients in Medicare Advantage excluded contracts* (N=1,290,777)	SMD
**Mean Age (SD)**	72.7	72.76	0.01
**Sex (Male)**			
Female	56.4%	56.8%	0.01
Male	43.6%	43.2%	0.01
**Race**			
Black	13.1%	9.7%	0.11
Hispanic	0.73%	1.15%	0.05
White	69.7%	66.1%	0.08
Other**	16.5%	23.1%	0.17
**Dual-eligible for Medicaid**	14.1%	20.2%	0.17
**Risk Score**	0.86	0.83	0.03
**Mortality**	3.43%	2.46%	0.06
**Region**			
Northeast	16.6%	20.8%	0.11
Midwest	20.6%	23.8%	0.08
South	43.9%	20.6%	0.50
West	18.9%	34.8%	0.38
**Area Deprivation Index**			
Lowest Quartile	24.1%	20.5%	0.09
2nd Lowest Quartile	15.1%	10.2%	0.14
3rd Lowest Quartile	5.0%	2.3%	0.13
Highest Quartile	0.3%	0.3%	0.00

*The study began with 718 MA contracts. 210 of them were highly reliable contracts, and 508 contracts were excluded (accounting for 38% of the MA patients, or 1,290,777 of the 3,956,117 patients).
**Other includes the American-Indian/Alaska Native, Asian/Pacific Islander, and “Other” categories from the Research Triangle Institute (RTI) Race Code.

Abbreviations: HCC, hierarchal condition category; MA, Medicare Advantage; SD, standard deviation; SMD, standardized mean difference (of which values <0.10 are generally considered negligible); TM, Traditional Medicare

© 2023 Beckman AL et al. *JAMA Health Forum.*

eTable 2. Event characteristics for Medicare Advantage contracts with highly reliable data versus Medicare Advantage excluded contracts

	**Highly Reliable MA Contracts Mean (SD)**	**Excluded (Less Reliable) MA Contracts Mean (SD)**	**% Difference**
**No. MA Contracts**	210	508	
**No. MA Beneficiaries**	13,426	3,427	
**Acute Care Visit Type**			
**Combined:**			
Overall	105.3 (535.4)	85.9 (488.7)	18.40%
Acute	40.6 (269.3)	32.5 (241.6)	19.90%
Chronic	64.7 (438.1)	53.4 (403.4)	17.50%
**Hospitalization:**			
Overall	30.2 (223.3)	21.5 (192.4)	28.70%
Acute	10.2 (110.7)	6.7 (87.7)	34.60%
Chronic	20 (185.7)	14.9 (164.4)	25.70%
**ED direct discharge:**			
Overall	61.1 (349.7)	53 (328.3)	13.20%
Acute	25.8 (190.6)	22.1 (179)	14.40%
Chronic	35.3 (279.7)	30.9 (262.3)	12.40%
**Observation stay:**			
Overall	14.0 (142.6)	11.3 (129.3)	18.80%
Acute	4.6 (74.2)	3.8 (67)	17.90%
Chronic	9.4 (118.6)	7.6 (108.3)	19.30%

© 2023 Beckman AL et al. *JAMA Health Forum.*

eTable 3. Differences in avoidable acute care episodes related to ambulatory care-sensitive conditions among Medicare Advantage versus Traditional Medicare using linear regression models

Acute Care Visit Type	Difference (MA vs. TM)	P-Value
**Hospitalization:**		
Overall	-4.40 [-4.74, -4.06]	<0.001
Acute	-3.27 [-3.45, -3.08]	<0.001
Chronic	-1.14 [-1.42, -0.85]	<0.001
**ED direct discharge:**		
Overall	17.74 [17.30, 18.18]	<0.001
Acute	4.89 [4.64, 5.15]	<0.001
Chronic	12.85 [12.50, 13.20]	<0.001
**Observation stay:**		
Overall	7.82 [7.66, 7.97]	<0.001
Acute	2.18 [2.10, 2.27]	<0.001
Chronic	5.64 [5.51, 5.76]	<0.001
**Combined:**		
Overall	21.16 [20.49, 21.84]	<0.001
Acute	3.81 [3.45, 4.18]	<0.001
Chronic	17.35 [16.80, 17.90]	<0.001

1) The sample for this analysis included Medicare Advantage (N=2,665,340) and Fee-for-Service (N=7,981,576) beneficiaries.

2) Differences reflect estimates from linear regression models. HCC scores were deflated by 6% in all models for all MA beneficiaries to account for more aggressive coding practices. Analyses have been adjusted for age, sex, dual status for Medicaid eligibility, self-reported race and ethnicity, the number of months alive for each beneficiary, HCC risk score, and include county-fixed effects.

3) For the 12 primary outcomes in this table, we used a Bonferroni adjustment, assessing for *P*-values less than 0.004. All coefficients met this threshold for statistical significance.

Abbreviations: HCC, hierarchal condition category; MA, Medicare Advantage; TM, Traditional Medicare

© 2023 Beckman AL et al. *JAMA Health Forum.*

eTable 4. Differences in avoidable acute care episodes related to ambulatory care-sensitive conditions among Medicare Advantage versus Traditional Medicare using 11% HCC deflation

Acute Care Visit Type	Adjusted RR (MA vs. TM)	P-Value
**Hospitalization:**		
Overall	1.00 [0.99, 1.01]	0.9488
Acute	0.89 [0.88, 0.90]	<0.001
Chronic	1.08 [1.06, 1.09]	<0.001
**ED direct discharge:**		
Overall	1.50 [1.49, 1.50]	<0.001
Acute	1.32 [1.30, 1.33]	<0.001
Chronic	1.67 [1.65, 1.68]	<0.001
**Observation stay:**		
Overall	2.50 [2.47, 2.54]	<0.001
Acute	2.16 [2.11, 2.21]	<0.001
Chronic	2.73 [2.68, 2.78]	<0.001
**Combined:**		
Overall	1.37 [1.36, 1.37]	<0.001
Acute	1.21 [1.21, 1.22]	<0.001
Chronic	1.49 [1.48, 1.50]	<0.001

1) The sample for this analysis included Medicare Advantage (N=2,665,340) and Fee-for-Service (N=7,981,576) beneficiaries.

2) Relative risks (RR) reflect estimates from Poisson models. HCC scores were deflated by 11% in all models for all MA beneficiaries to account for more aggressive coding practices. Analyses have been adjusted for age, sex, dual status for Medicaid eligibility, self-reported race and ethnicity, the number of months alive for each beneficiary, HCC risk score, and include county-fixed effects.

3) For the 12 primary outcomes in this table, we used a Bonferroni adjustment, assessing for *P*-values less than 0.004. All coefficients met this threshold for statistical significance except for the measure of overall hospitalizations.

Abbreviations: HCC, hierarchal condition category; MA, Medicare Advantage; TM, Traditional Medicare

© 2023 Beckman AL et al. *JAMA Health Forum.*

eTable 5. Differences in avoidable acute care episodes related to ambulatory care-sensitive conditions among MA HMOs versus MA PPOs using linear regression models

Acute Care Visit Type	Differences (HMO vs. PPO)	P-Value
**Hospitalization:**		
Overall	-1.58 [-2.21, -0.95]	<0.001
Acute	-1.54 [-1.87, -1.21]	<0.001
Chronic	-0.04 [-0.57, 0.49]	0.8917
**ED direct discharge:**		
Overall	3.52 [2.49, 4.55]	<0.001
Acute	0.75 [0.18, 1.32]	0.0096
Chronic	2.77 [1.94, 3.60]	<0.001
**Observation stay:**		
Overall	1.19 [0.77, 1.62]	<0.001
Acute	0.27 [0.04, 0.49]	0.0192
Chronic	0.93 [0.57, 1.28]	<0.001
**Combined:**		
Overall	3.13 [1.61, 4.65]	<0.001
Acute	-0.52 [-1.32, 0.27]	0.1960
Chronic	3.66 [2.39, 4.92]	<0.001

1) The sample for this analysis included HMO (N=2,392,967) and PPO (N=1,334,958) beneficiaries.

2) Differences reflect estimates from linear regression models. Analyses have been adjusted for age, sex, dual status for Medicaid eligibility, self-reported race and ethnicity, the number of months alive for each beneficiary, HCC risk score, and include county-fixed effects

3) For the 12 primary outcomes in this table, we used a Bonferroni adjustment, assessing for *P*-values less than 0.004. All coefficients met this threshold for statistical significance besides hospitalizations for chronic conditions, ED direct discharges for acute conditions, observation stays for acute conditions, and combined visits for acute conditions.

Abbreviations: HMO, Health Maintenance Organization; MA, Medicare Advantage; PPO, Preferred Provider Organization; TM, Traditional Medicare

eTable 6. Differences in avoidable acute care episodes related to ambulatory care-sensitive conditions among MA high-quality versus MA lower-quality plans

Acute Care Visit Type	Adjusted RR (4-5 star vs. 1-3 star)	P-Value
**Combined:**		
Overall	0.95 [0.93, 0.96]	<0.001
Acute	0.96 [0.94, 0.98]	<0.001
Chronic	0.94 [0.92, 0.95]	<0.001
**Hospitalization:**		
Overall	0.95 [0.93, 0.97]	<0.001
Acute	1.01 [0.98, 1.04]	0.6391
Chronic	0.92 [0.90, 0.94]	<0.001
**ED direct discharge:**		
Overall	0.92 [0.90, 0.93]	<0.001
Acute	0.93 [0.91, 0.95]	<0.001
Chronic	0.91 [0.89, 0.93]	<0.001
**Observation stay:**		
Overall	1.06 [1.03, 1.09]	<0.001
Acute	1.06 [1.01, 1.12]	0.0298
Chronic	1.06 [1.02, 1.10]	0.0028

1) The sample for this analysis included beneficiaries in MA High-Quality Plans (N=1,878,048) and MA Lower-Quality Plans (N=520,015).

2) Relative risks (RR) reflect estimates from Poisson models. HCC scores were deflated by 6% in all models for all MA beneficiaries to account for more aggressive coding practices. Analyses have been adjusted for age, sex, dual status for Medicaid eligibility, self-reported race and ethnicity, the number of months alive for each beneficiary, HCC risk score, and include Hospital Referral Region-fixed effects.

3) For the 12 primary outcomes in this table, we used a Bonferroni adjustment, assessing for *P*-values less than 0.004. All coefficients met this threshold for statistical significance besides hospitalizations for acute conditions and observation stays for acute conditions.

Abbreviations: MA, Medicare Advantage; TM, Traditional Medicare

© 2023 Beckman AL et al. *JAMA Health Forum.*

eTable 7. Differences in avoidable acute care episodes related to ambulatory care-sensitive conditions among Medicare Advantage versus Traditional Medicare using Poisson models, limiting to counties with 100+ patients

Acute Care Visit Type	Adjusted RR (MA vs. TM)	P-Value
**Combined:**		
Overall	1.30 [1.30, 1.31]	<0.001
Acute	1.17 [1.16, 1.18]	<0.001
Chronic	1.41 [1.40, 1.42]	<0.001
**Hospitalization:**		
Overall	0.94 [0.93, 0.95]	<0.001
Acute	0.84 [0.83, 0.85]	<0.001
Chronic	1.00 [1.00, 1.01]	0.2878
**ED direct discharge:**		
Overall	1.44 [1.43, 1.45]	<0.001
Acute	1.27 [1.26, 1.29]	<0.001
Chronic	1.60 [1.59, 1.62]	<0.001
**Observation stay:**		
Overall	2.38 [2.35, 2.41]	<0.001
Acute	2.07 [2.02, 2.12]	<0.001
Chronic	2.58 [2.54, 2.63]	<0.001

1) 122 out of 3256 counties have less than 100 Medicare beneficiaries, which account for 4765 beneficiaries. After exclusions, the total sample for this analysis included Medicare Advantage (N=2,664,648) and Fee-for-Service (N=7,961,236) beneficiaries.

2) Relative risks (RR) reflect estimates from Poisson models, accounting for overdispersion. HCC scores were deflated by 6% in all models for all MA beneficiaries to account for more aggressive coding practices. Analyses have been adjusted for age, sex, dual status for Medicaid eligibility, self-reported race and ethnicity, the number of months alive for each beneficiary, HCC risk score, and include county-fixed effects.

3) For the 12 primary outcomes in this table, we used a Bonferroni adjustment, assessing for *P*-values less than 0.004. All coefficients met this threshold for statistical significance besides hospitalizations for chronic conditions.

Abbreviations: HCC, hierarchal condition category; MA, Medicare Advantage; TM, Traditional Medicare

© 2023 Beckman AL et al. *JAMA Health Forum.*

eTable 8. Differences in avoidable acute care episodes related to ambulatory care-sensitive conditions among Medicare Advantage versus Traditional Medicare using Poisson models, accounting for overdispersion

Acute Care Visit Type	Adjusted RR (MA vs. TM)	P-Value
**Combined:**		
Overall	1.30 [1.30, 1.31]	<.0001
Acute	1.17 [1.16, 1.18]	<.0001
Chronic	1.41 [1.40, 1.42]	<.0001
**Hospitalization:**		
Overall	0.94 [0.93, 0.95]	<.0001
Acute	0.84 [0.83, 0.85]	<.0001
Chronic	1.00 [1.00, 1.01]	0.2772
**ED direct discharge:**		
Overall	1.44 [1.43, 1.45]	<.0001
Acute	1.27 [1.26, 1.29]	<.0001
Chronic	1.60 [1.59, 1.62]	<.0001
**Observation stay:**		
Overall	2.38 [2.35, 2.41]	<.0001
Acute	2.07 [2.02, 2.12]	<.0001
Chronic	2.58 [2.54, 2.63]	<.0001

1) The sample for this analysis included Medicare Advantage (N=2,665,340) and Fee-for-Service (N=7,981,576) beneficiaries.

2) Relative risks (RR) reflect estimates from Poisson models, accounting for overdispersion. HCC scores were deflated by 6% in all models for all MA beneficiaries to account for more aggressive coding practices. Analyses have been adjusted for age, sex, dual status for Medicaid eligibility, self-reported race and ethnicity, the number of months alive for each beneficiary, HCC risk score, and include county-fixed effects.

3) For the 12 primary outcomes in this table, we used a Bonferroni adjustment, assessing for *P*-values less than 0.004. All coefficients met this threshold for statistical significance besides hospitalizations for chronic conditions.

Abbreviations: HCC, hierarchal condition category; MA, Medicare Advantage; TM, Traditional Medicare

© 2023 Beckman AL et al. *JAMA Health Forum.*

eTable 9. Differences in avoidable acute care episodes related to ambulatory care-sensitive conditions among Medicare Advantage versus Traditional Medicare using Poisson models, accounting for overdispersion and with months alive as offset

Acute Care Visit Type	Adjusted RR (MA vs. TM)	P-Value
**Combined:**		
Overall	1.25 [1.25, 1.26]	<0.001
Acute	1.12 [1.11, 1.13]	<0.001
Chronic	1.36 [1.35, 1.37]	<0.001
**Hospitalization:**		
Overall	0.88 [0.87, 0.89]	<0.001
Acute	0.79 [0.78, 0.80]	<0.001
Chronic	0.94 [0.93, 0.95]	<0.001
**ED direct discharge:**		
Overall	1.42 [1.41, 1.43]	<0.001
Acute	1.25 [1.23, 1.26]	<0.001
Chronic	1.58 [1.56, 1.59]	<0.001
**Observation stay:**		
Overall	2.34 [2.31, 2.37]	<0.001
Acute	2.03 [1.98, 2.07]	<0.001
Chronic	2.55 [2.51, 2.59]	<0.001

1) The sample for this analysis included Medicare Advantage (N=2,665,340) and Fee-for-Service (N=7,981,576) beneficiaries.

2) Relative risks (RR) reflect estimates from Poisson models, accounting for overdispersion. HCC scores were deflated by 6% in all models for all MA beneficiaries to account for more aggressive coding practices. Analyses have been adjusted for age, sex, dual status for Medicaid eligibility, self-reported race and ethnicity, HCC risk score, and include county-fixed effects.

3) For the 12 primary outcomes in this table, we used a Bonferroni adjustment, assessing for *P*-values less than 0.004. All coefficients met this threshold for statistical significance.

Abbreviations: HCC, hierarchal condition category; MA, Medicare Advantage; TM, Traditional Medicare

© 2023 Beckman AL et al. *JAMA Health Forum.*

eTable 10. Differences in avoidable acute care episodes related to ambulatory care-sensitive conditions among Medicare Advantage versus Traditional Medicare using Poisson models, accounting for overdispersion and without HCC adjustment

Acute Care Visit Type	Adjusted RR (MA vs. TM)	P-Value
**Combined:**		
Overall	1.12 [1.12, 1.13]	<0.001
Acute	1.04 [1.03, 1.05]	<0.001
Chronic	1.19 [1.18, 1.20]	<0.001
**Hospitalization:**		
Overall	0.76 [0.75, 0.76]	<0.001
Acute	0.71 [0.70, 0.72]	<0.001
Chronic	0.79 [0.78, 0.80]	<0.001
**ED direct discharge:**		
Overall	1.31 [1.30, 1.32]	<0.001
Acute	1.17 [1.16, 1.18]	<0.001
Chronic	1.43 [1.41, 1.45]	<0.001
**Observation stay:**		
Overall	2.05 [2.02, 2.08]	<0.001
Acute	1.83 [1.79, 1.88]	<0.001
Chronic	2.19 [2.15, 2.23]	<0.001

1) The sample for this analysis included Medicare Advantage (N=2,665,340) and Fee-for-Service (N=7,981,576) beneficiaries.

2) Relative risks (RR) reflect estimates from Poisson models, accounting for overdispersion. Analyses have been adjusted for age, sex, dual status for Medicaid eligibility, self-reported race and ethnicity, the number of months alive for each beneficiary, and include county-fixed effects.

3) For the 12 primary outcomes in this table, we used a Bonferroni adjustment, assessing for *P*-values less than 0.004. All coefficients met this threshold for statistical significance.

Abbreviations: HCC, hierarchal condition category; MA, Medicare Advantage; TM, Traditional Medicare

© 2023 Beckman AL et al. *JAMA Health Forum.*

eTable 11. Differences in avoidable acute care episodes related to ambulatory care-sensitive conditions among Medicare Advantage versus Traditional Medicare using Poisson models, stratified by geographic region

Acute Care Visit Type	Northeast		Midwest		South		West	
	Adjusted RR (MA vs. TM)	P-Value	Adjusted RR (MA vs. TM)	P-Value	Adjusted RR (MA vs. TM)	P-Value	Adjusted RR (MA vs. TM)	P-Value
**Combined:**								
Overall	1.31 [1.29, 1.32]	<0.001	1.23 [1.21, 1.24]	<0.001	1.28 [1.27, 1.30]	<0.001	1.24 [1.22, 1.26]	<0.001
Acute	1.16 [1.14, 1.18]	<0.001	1.10 [1.08, 1.12]	<0.001	1.14 [1.12, 1.15]	<0.001	1.13 [1.11, 1.15]	<0.001
Chronic	1.42 [1.39, 1.44]	<0.001	1.33 [1.31, 1.35]	<0.001	1.40 [1.39, 1.42]	<0.001	1.34 [1.31, 1.37]	<0.001
**Hospitalization:**								
Overall	0.93 [0.92, 0.95]	<0.001	0.95 [0.94, 0.97]	<0.001	0.95 [0.94, 0.96]	<0.001	0.81 [0.80, 0.83]	<0.001
Acute	0.82 [0.79, 0.84]	<0.001	0.85 [0.83, 0.87]	<0.001	0.84 [0.82, 0.86]	<0.001	0.75 [0.72, 0.78]	<0.001
Chronic	1.00 [0.98, 1.02]	0.9202	1.02 [1.00, 1.04]	0.0184	1.02 [1.01, 1.03]	0.0037	0.86 [0.84, 0.88]	<0.001
**ED direct discharge:**								
Overall	1.52 [1.49, 1.55]	<0.001	1.30 [1.28, 1.32]	<0.001	1.39 [1.38, 1.41]	<0.001	1.37 [1.34, 1.39]	<0.001
Acute	1.31 [1.27, 1.34]	<0.001	1.17 [1.14, 1.19]	<0.001	1.22 [1.21, 1.24]	<0.001	1.23 [1.20, 1.26]	<0.001
Chronic	1.73 [1.68, 1.77]	<0.001	1.43 [1.40, 1.46]	<0.001	1.54 [1.52, 1.57]	<0.001	1.50 [1.47, 1.54]	<0.001
**Observation stay:**								
Overall	2.50 [2.41, 2.59]	<0.001	2.20 [2.14, 2.26]	<0.001	2.32 [2.27, 2.36]	<0.001	2.29 [2.21, 2.37]	<0.001
Acute	2.19 [2.06, 2.33]	<0.001	1.90 [1.81, 1.98]	<0.001	2.04 [1.97, 2.11]	<0.001	1.83 [1.72, 1.94]	<0.001
Chronic	2.73 [2.61, 2.85]	<0.001	2.41 [2.32, 2.49]	<0.001	2.50 [2.44, 2.56]	<0.001	2.63 [2.52, 2.75]	<0.001

Notes:
1) The sample for this analysis included Medicare Advantage (N=2,665,340) and Fee-for-Service (N=7,981,576) beneficiaries.
2) Relative risks (RR) reflect estimates from Poisson models. HCC scores were deflated by 6% in all models for all MA beneficiaries to account for more aggressive coding practices. Analyses have been adjusted for age, sex, dual status for Medicaid eligibility, self-reported race and ethnicity, the number of months alive for each beneficiary, HCC risk score, and include county-fixed effects.
3) For the 48 outcomes in this table, we used a Bonferroni adjustment, assessing for *P*-values less than 0.001. All coefficients met this threshold for statistical significance except for the following: hospitalizations for chronic conditions in the Northeast, Midwest, and South.

Abbreviations: HCC, hierarchal condition category; MA, Medicare Advantage; TM, Traditional Medicare

© 2023 Beckman AL et al. *JAMA Health Forum.*

eTable 12. Discharge location after ED direct discharge or observation stay for Medicare Advantage versus Traditional Medicare

Discharge location	Traditional Medicare (% of patients)	Medicare Advantage (% of patients)
Home or self-care	91.0%	93.1%
Transfer	0.5%	0.5%
Skilled nursing facility	0.4%	0.4%
Home with home health services	0.2%	0.3%
Expired	0.06%	0.05%
Rehabilitation facility	0.04%	0.03%
Other	7.7%	5.6%
Total	100.0%	100.0%

The sample for this analysis included Medicare Advantage (N=2,665,340) and Fee-for-Service (N=7,981,576) beneficiaries.

© 2023 Beckman AL et al. *JAMA Health Forum.*
