# Supplementary material for: Evaluation of Potentially Avoidable Acute Care Utilization Among Patients Insured by Medicare Advantage vs Traditional Medicare
Source: JAMA Health Forum. 2023 Feb 24;4(2):e225530. doi: 10.1001/jamahealthforum.2022.5530 (PMC9958527; doi:10.1001/jamahealthforum.2022.5530)
Supplement: Supplement 2. — Data Sharing Statement [file jamahealthforum-e225530-s002.pdf]

Data Sharing Statement

Beckman. Evaluation of Potentially Avoidable Acute Care Utilization Among Patients Insured by Medicare Advantage vs Traditional Medicare. *JAMA Health Forum*. Published February 24, 2023. doi:10.1001/jamahealthforum.2022.5530

Data
Data available: No

Additional Information
Explanation for why data not available: Medicare research-identifiable files may not be shared due to restrictions made by Medicare in our data use agreement. However, any researcher interested in Medicare data can obtain these data directly through Medicare and ResDAC.
